# Childhood maltreatment and emotion regulation in everyday life: an experience sampling study

**DOI:** 10.1038/s41598-023-34302-9

**Published:** 2023-05-03

**Authors:** Andrei Ion, Mirela I. Bîlc, Simina Pițur, Claudia Felicia Pop, Aurora Szentágotai-Tătar, Andrei C. Miu

**Affiliations:** 1 Assessment and Individual Differences – AID Lab, Department of Psychology and Cognitive Science, University of Bucharest, Bucharest, Romania; 2 Cognitive Neuroscience Laboratory, Department of Psychology, Babeș-Bolyai University, 37 Republicii, 400015 Cluj-Napoca, CJ Romania; 3 Institute of Medical Psychology, Medical Faculty, Otto-Von-Guericke University of Magdeburg, Magdeburg, Germany; 4 Nursing Discipline, Department Mother and Child, University of Medicine and Pharmacy Iuliu Haţieganu, Cluj-Napoca, Romania; 5 Department of Clinical Psychology and Psychotherapy, Babeș-Bolyai University, Cluj-Napoca, Romania

**Keywords:** Psychology, Human behaviour

## Abstract

Childhood maltreatment is a major risk factor for psychopathology, and increasing evidence suggests that emotion regulation is one of the underlying mechanisms. However, most of this evidence comes from single assessments of habitual emotion regulation, which may not overlap with spontaneous emotion regulation in daily life and which fail to account for within-individual variability in emotion regulation across multiple contexts. In the present study, we investigated the relation between history of childhood maltreatment, positive and negative affect, and multiple dimensions of spontaneous emotion regulation (strategy use, emotion regulation goals, emotion regulation success and effort) in everyday life, using experience sampling method (3 assessments/day, for 10 consecutive days), in a sample of healthy volunteers (N = 118). Multilevel modeling results indicated that childhood maltreatment was associated with lower positive affect and higher negative affect. Childhood maltreatment was also related to lower use of reappraisal and savoring (but not suppression, rumination and distraction), reduced emotion regulation success (but not effort), as well as lower levels of and higher within-individual variability of hedonic (but not instrumental) emotion regulation goals. These results provide ecological evidence for multiple differences in emotion regulation in individuals with a history of childhood maltreatment.

## Introduction

Childhood maltreatment is associated with increased lifelong risk for virtually all common mental disorders^1–3^. Recent efforts have focused on uncovering the psychological mechanisms underlying the influence of childhood maltreatment on vulnerability to psychopathology^4–6^. This issue has major translational implications, especially considering that individuals with a history of childhood maltreatment may show reduced response to current mental health interventions^7^. Identifying the mechanisms underlying the pervasive impact of childhood maltreatment will contribute to increasing the specificity of psychological interventions in this vulnerable category of the population.

### Childhood maltreatment and emotion regulation

Emotion regulation (i.e., the processes by which one attempts to regulate the frequency and intensity of emotion^8^) is one of the candidate mechanisms that has been extensively examined in relation to maltreatment and psychopathology. An important argument supporting this hypothesis is that emotion regulation problems have been documented in multiple forms of psychopathology^9^, and may thus explain the transdiagnostic risk associated with childhood maltreatment. Furthermore, the protracted development of emotion regulation, which spans childhood and adolescence, may make it particularly vulnerable to the detrimental effects of maltreatment occurring during these developmental periods^10^.

Previous studies have generally supported this view^11–13^, but their focus on multiple dimensions of emotion regulation and the heterogeneity of theoretical approaches have made it difficult, until recently, to draw a firm conclusion. A large-scale meta-analysis has examined the relation between childhood maltreatment and the habitual use of multiple emotion regulation strategies including cognitive reappraisal (i.e., reinterpreting an event so as to modulate its emotional impact), rumination (i.e., dwelling on an emotion, the situation that triggered it or its consequences), expressive suppression (i.e., blocking the expression of emotion) and distraction (i.e., moving attention away from an emotional event, by thinking about something else or engaging in another activity)^13^. Results have indicated that maltreatment is consistently associated with reduced habitual reappraisal, and increased habitual rumination and expressive suppression. Furthermore, these differences in emotion regulation have shown a consistent mediator role in the relation between childhood maltreatment and symptoms of psychopathology^13^.

### Moving from habitual to spontaneous emotion regulation

These results^13^ warrant further work in this area, while also drawing attention to the limitations of the current literature. Until now, the large majority of studies have relied on single retrospective assessments of the habitual use of emotion regulation (i.e., global, trait-like assessments of the strategies that are typically used), which, on the one hand, are susceptible to recall limitations and generalization biases, and, on the other, fail to account for within-individual variability. People draw on different knowledge when reporting global (e.g., how you think you are in general) versus momentary (e.g., how you think you are at present) characteristics of emotion^14^, and there is evidence that reports of the habitual use of emotion regulation strategies have low ecological and discriminant validity in relation to the spontaneous use of those strategies in daily life^15–17^. For example, self-reported habitual reappraisal predicts not only reappraisal use in daily life, but also rumination and suppression use^16^. Furthermore, habitual measures may also reflect other aspects of emotion regulation, such as its success: the self-reported frequency of using reappraisal "in general", for instance, shows a positive, albeit modest association with the efficiency of using reappraisal to modulate emotion in laboratory tasks^18,19^. Overall, evidence suggests that habitual measures poorly predict momentary measures of emotion regulation, and the former may capture differences in multiple stages of emotion regulation (e.g., selection, implementation). Failing to account for within-individual variability is another limit of previous studies considering that there is considerable emotion regulation variability over contexts and time^20,21^.

Experience sampling method (ESM) offers an alternative approach, which has been increasingly used in studies on emotion and psychopathology^22,23^. In addition to capitalizing on ecological validity, this approach involves multiple assessments of recent experience, with the obvious advantages of reducing recall biases and of accounting for both within-, and between-individual variance^24^. However, to date, there is a dearth of evidence on childhood maltreatment and emotion and emotion regulation using this approach. For example, several studies have employed ESM to investigate the impact of childhood adversity on negative and positive affect, with heterogeneous results. Some^25–27^, but not all studies^28–30^ found increased negative affect in individuals with a history of childhood maltreatment. Similarly, some studies reported lower levels^25^ or higher variability^30^ of positive affect in childhood maltreatment, while other studies failed to replicate this association^27,29^. In which emotion regulation is concerning, there is very limited data from field studies. To our knowledge, there is only one diary study^31^ which assessed expressive suppression in relation to stressful events that happened throughout the day, for seven consecutive days. Results indicated that history of emotional abuse was associated with increased use of suppression, particularly in individuals with lower vagal flexibility and higher attentional capacity^31^.

### The present study

#### Childhood maltreatment, affect and emotion regulation strategies

The present study used ESM to assess positive and negative affect, as well as the use of multiple emotion regulation strategies (i.e., reappraisal, distraction, savoring, rumination, suppression) three times a day, for ten consecutive days, in a sample of healthy volunteers in which history of childhood maltreatment was characterized. In line with previous ESM studies on positive and negative affect^25^, we expected that childhood maltreatment would be associated with increased negative affect and decreased positive affect. In addition, we predicted, based on our recent meta-analysis^13^ (but also mindful of the differences between habitual measures of emotion regulation, and spontaneous emotion regulation in daily life^16,17^), that childhood maltreatment would be associated with reduced use of reappraisal, and increased use of rumination and suppression. We also assessed savoring, an emotion regulation strategy typically aimed at enhancing positive emotions^32^, and expected that the use of this strategy was negatively associated with maltreatment. In which distraction was concerned, we did not have a hypothesis, considering that this strategy has proven to be adaptive in some, but not all contexts^33^, and meta-analytic results on habitual distraction failed to show a consistent association with childhood maltreatment^13^. Notably, we examined the relation between history of childhood maltreatment and differences in the use of emotion regulation strategies, characterized at both the within-individual (i.e., variation in the use of a strategy from one situation to another) and the between-individual (i.e., variation in the use of a strategy across contexts, from one person to another) level.

#### Childhood maltreatment and emotion regulation variability

Recent perspectives on emotion regulation have emphasized that no single emotion regulation strategy is efficient in all situations, and that future efforts should focus on emotion regulation flexibility or the capacity to adapt emotion regulation to situational demands^34–36^. Several studies have shown that reduced flexibility in expressing and suppressing emotions (i.e., switching from one behavior to the other, as demanded by the situation), for instance, is associated with poor mental health^37–39^. Using similar laboratory measures, we have also found that people with a history of childhood maltreatment show reduced expressive flexibility^40^. However, the topic of emotion regulation flexibility has been examined relatively little in spontaneous emotion regulation and everyday life.

A recent ESM study^41^ proposed an approach to studying emotion regulation variability (i.e., a superordinate concept relative to flexibility) by using standard deviations of the distribution of emotion regulation measures in order to estimate between-strategy variability (i.e., variability in the level of employing multiple strategies at one occasion) and within-strategy variability (i.e., variability in the level of employing a single strategy across occasions). The results of this study^41^ suggested that variability in the emotion regulation strategies that are employed in the same situation may be adaptive, considering its association with reduced negative affect.

In keeping with this study, we examined the relations between childhood maltreatment and emotion regulation variability in the present analyses. Given the breadth of emotion regulation measures in the present study and the novelty of the topic, we also investigated the links between emotion regulation variability and affect, emotion regulation goals, and emotion regulation success and effort.

#### Childhood maltreatment and emotion regulation goals

Another aim of this study was to examine the relations between childhood maltreatment and emotion regulation goals. According to the process model of emotion regulation^8^, the activation of an emotion regulation goal (i.e., why we choose to regulate emotion) is the starting point of emotion regulation efforts. Emotion regulation goals have generally been categorized into hedonic (e.g., regulating emotions so as to feel better) and instrumental (e.g., regulating emotions so as to facilitate performance and maintain social connections)^42^. Evidence suggests that hedonic goals are associated with the use of strategies such as reappraisal and distraction, but not suppression, and that instrumental goals are associated with the use of reappraisal^43^. However, to our knowledge, no study until now has investigated whether history of childhood maltreatment is related to differences in emotion regulation goals. We propose two alternative hypotheses, according to which maltreatment could be associated either with lower levels of hedonic emotion regulation goals (i.e., a hypothesis consistent with anhedonia in maltreatment and psychopathology^44,45^) or with higher levels of hedonic motives (i.e., an alternative hypothesis consistent with exaggerated self-focus^46,47^).

#### Childhood maltreatment and emotion regulation success and effort

The present study also investigated the relation between childhood maltreatment and emotion regulation success and effort. An extensive literature indicates that maltreatment is associated with reduced self-reported efficiency in emotion regulation, as reflected by perceived difficulty in controlling cognitive performance and social behavior in the presence of emotion^13^. However, all previous studies have used global assessments of emotion regulation success, and single measures. Therefore, it remains to be examined whether multiple momentary assessments of emotion regulation success, as employed in the present ESM study, paint a similar picture, and whether differences reside at the between- or within-individual level. Finally, evidence of increased prefrontal activity during reappraisal in childhood maltreatment^48^ were taken to suggest that people with a history of maltreatment may employ additional effort in emotion regulation. In the present study, we investigated this hypothesis based on self-reports of effort in emotion regulation.

## Results

### Descriptive and preliminary analyses

Table 1 shows the childhood maltreatment scores and their distribution into severity categories. Overall, 58.50% of participants had scores above the low-to-moderate threshold for at least one type of maltreatment.

**Table 1:** Childhood maltreatment scores and severity categories.

CTQ subscale	Mean score (± SEM)	Severity categories (frequency and %)			
		None (or minimal)	Low to moderate	Moderate to severe	Severe to extreme
Emotional abuse	9.43 ± 4.75	67 (56.78)	29 (24.58)	8 (6.78)	14 (11.86)
Physical abuse	6.43 ± 2.71	99 (83.90)	10 (8.47)	3 (2.54)	6 (5.08)
Sexual abuse	5.64 ± 2.26	102 (86.44)	7 (5.93)	4 (3.39)	5 (4.24)
Emotional neglect	10.01 ± 4.56	67 (56.78)	26 (22.03)	16 (13.56)	9 (7.63)
Physical neglect	6.16 ± 2.19	100 (84.75)	9 (7.63)	6 (5.08)	3 (2.54)

CTQ, Childhood Trauma Questionnaire; SEM, standard error of the mean.

In the ESM, the average questionnaire completion rate was 88.90%. No incomplete entries (participants completing only part of the survey on any given occasion) were observed. The number of occasions when participants failed to fill in the questionnaire ranged between 0 and 13, with an average of 3.33 occasions. The intra-class correlations (ICCs), which indicate the proportion of variance at the between-individual level, ranged between 0.16 for savoring, and 0.52 for instrumental emotion regulation goals, with a mean of 0.32 across measures. This suggests that there was variance in affect and emotion regulation, at both the between- and the within-individual level (see Tables 2, 3, 5, 6).

**Table 2:** Within-person and between-person relations between childhood maltreatment and negative and positive affect.

Dimension	Parameter	Within-individual level			Between-individual level		
		Unstandardized estimate	SE	*p*	Unstandardized estimate	SE	*p*
Negative affect (ICC = 0.37)	Intercept	–	–	–	3.08***	0.22	0.000
	CTQ Total	0.01	0.01	0.680	0.25**	0.26	0.006
	Residual variance	0.99***	0.00	0.000	0.94***	0.04	0.000
	*R*^2^	0.00	0.00	0.837	0.00	0.00	0.907
Positive affect (ICC = 0.27)	Intercept	–	–	–	3.08**	0.09	0.050
	CTQ Total	−0.01	0.02	0.814	−0.19***	0.09	0.000
	Residual variance	0.99***	0.00	0.000	0.97***	0.03	0.000
	*R*^2^	0.00	0.00	0.987	0.03	0.05	0.326

*p < .05. **p < .01. ***p < .001.

CTQ, Childhood Trauma Questionnaire; ICC, intra-class correlation; SE, standard error.

**Table 3:** Within-individual and between-individual relations between childhood maltreatment and emotion regulation strategies.

Dimension	Parameter	Within-individual level			Between-individual level		
		unstandardized estimate	SE	*p*	unstandardized estimate	SE	*p*
Suppression (ICC = 0.28)	Intercept	–	–	–	3.08***	0.22	0.000
	CTQ Total	0.02	0.03	0.198	0.02	0.10	0.856
	Residual variance	0.99***	0.00	0.000	0.99***	0.01	0.000
	*R*^2^	0.00	0.00	0.520	0.00	0.00	0.927
Distraction (ICC = 0.28)	Intercept	–	–	–	3.08***	0.22	0.000
	CTQ Total	0.02	0.02	0.344	0.02	0.10	0.800
	Residual variance	0.99	0.00	0.000	0.99***	0.01	0.000
	*R*^2^	0.00	0.00	0.636	0.00	0.01	0.899
Savoring (ICC = 0.16)	Intercept	–	–	–	3.08***	0.22	0.000
	CTQ Total	− 0.02	0.02	0.266	–0.28**	0.09	0.005
	Residual variance	0.99***	0.00	0.000	0.92***	0.05	0.000
	*R*^2^	0.00	0.00	0.578	0.07	0.05	0.159
Reappraisal (ICC = 0.32)	Intercept	–	–	–	3.08***	0.22	0.000
	CTQ Total	0.05	0.02	0.790	−0.16**	0.09	0.005
	Residual variance	0.99***	0.00	0.000	0.97***	0.03	0.000
	*R*^2^	0.00	0.00	0.894	0.03	0.03	0.395
Rumination (ICC = 0.31)	Intercept	–	–	–	3.08***	0.22	0.000
	CTQ Total	0.01	0.02	0.663	−0.06	0.10	0.529
	Residual variance	0.99***	0.00	0.000	0.99***	0.01	0.000
	*R*^2^	0.00	0.00	0.898	0.01	0.01	0.753

*p < .05. **p < .01. ***p < .001.

CTQ, Childhood Trauma Questionnaire; ICC, intra-class correlation; SE, standard error.

### Relations between emotion regulation and affect

We examined the relations between emotion regulation strategies, and between emotion regulation strategies, on the one hand, and affect, emotion regulation goals, and emotion regulation success and effort, on the other hand. All results are described in Supplementary Tables 1–4.

#### Relations among emotion regulation strategies

We first analyzed the associations between emotion regulation strategies (see Supplementary Table 1). At the between-individual level, there were significant positive associations between all strategies, with the exception of savoring, which did not significantly correlate with any of the other strategies. That is, individuals tended to use similar levels of reappraisal, distraction, rumination and suppression, but the use of savoring was independent of the other emotion regulation strategies. At the within-individual level, there were again significant positive associations between suppression, distraction, reappraisal and rumination. Savoring correlated negatively with the use of these strategies at the within-individual level. These results suggest that in situations in which reappraisal, distraction, rumination and suppression were used at higher levels, savoring was used at lower levels.

#### Relations between emotion regulation strategies and affect

The use of suppression, distraction, and rumination was positively associated with negative affect at the between-individual level (see Supplementary Table 2). In a complementary fashion, reappraisal and savoring were positively associated with positive affect at the between-individual level. That is, individuals who used higher levels of suppression, distraction and rumination reported higher levels of negative affect, whereas those who use reappraisal and savoring reported higher levels of positive affect. At the within-individual level, suppression and rumination, as well as distraction and reappraisal, were positively associated with negative affect, whereas savoring was negatively associated with negative affect. In other words, in situations in which suppression and rumination, distraction and reappraisal were increasingly used, and in which savoring was used at lower levels, there was more negative affect. At the within-individual level, savoring and suppression were positively associated, and distraction and rumination were negatively associated with positive affect. Therefore, higher levels of positive affect were reported in situations in which savoring and suppression were used at higher levels, and distraction and rumination at lower levels.

#### Relation between emotion regulation strategies and emotion regulation goals

There were also significant associations between the use of emotion regulation strategies and emotion regulation goals (see Supplementary Table 3). At the between-individual level, suppression, distraction, and rumination were positively associated with instrumental goals. Reappraisal was positively associated with hedonic goals. That is, individuals who reported using suppression, distraction and rumination at higher levels also reported that their emotion regulation efforts were increasingly motivated by performance-related and social goals, for instance. Those who used higher levels of reappraisal reported that their emotion regulation efforts were motivated by feeling better. At the within-individual level, the use of suppression, distraction, reappraisal, and rumination was positively associated with instrumental goals. Distraction, savoring, reappraisal, and rumination were positively associated with hedonic goals. This suggests that situations in which emotion regulation efforts were motivated by either instrumental or hedonic goals did not differ in terms of the emotion regulation strategies that were used, with the sole exception of suppression, which seemed to be specifically associated with performance or social goals.

#### Relation between emotion regulation strategies and emotion regulation success and effort

At the between-individual level, the use of suppression and distraction was positively associated, and the use of rumination was negatively associated with the perceived success of emotion regulation (see Supplementary Table 4). The use of distraction and rumination was positively associated with emotion regulation effort, also at the between-individual level. Overall, those who used suppression and distraction reported better success in regulating emotions, in contrast to those who use rumination. People who used higher levels or distraction and rumination reported that emotion regulation was more taxing. At the within-individual level, suppression, distraction, savoring and reappraisal were positively associated, and rumination was negatively associated with emotion regulation success. Suppression, distraction, reappraisal, and rumination were positively associated, and savoring was negatively associated with emotion regulation effort, also at the within-individual level. These results suggested that in situations that involved the use of any of the strategies except rumination, emotion regulation was perceived as more efficient. Those which involved the use of any of the strategies except savoring were perceived as more effortful.

### Childhood maltreatment, affect and emotion regulation

#### Childhood maltreatment and affect

**Figure 1:**
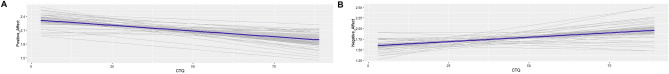
The association between childhood maltreatment and positive affect (**A**) and negative affect (**B**). CTQ, Childhood Trauma Questionnaire.

At the between-individual level, childhood maltreatment was significantly related to both positive affect and negative affect (Table 2). As expected, participants with higher levels of childhood maltreatment reported lower positive affect and higher negative affect (Fig. 1). At the within-individual level, the relations between childhood maltreatment and both positive and negative affect were not significant.

#### Childhood maltreatment and emotion regulation strategies

**Figure 2:**
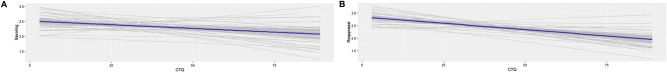
The association between childhood maltreatment savoring (**A**) and reappraisal (**B**). CTQ, Childhood Trauma Questionnaire.

At the between-individual level, significant negative associations were observed between childhood maltreatment and both savoring and reappraisal (Table 3). In other words, people with higher levels of maltreatment reported using less savoring and reappraisal (Fig. 2). No significant associations at the between-individual level were observed between childhood maltreatment and suppression, distraction and rumination. At the within-individual level, no significant relationships were found.

#### Childhood maltreatment and emotion regulation variability

We also analyzed the associations between childhood maltreatment and indices of emotion regulation variability (i.e., between- and within-strategy variability). Maltreatment was not significantly associated with between-strategy variability (*r* = 0.01, p = 0.242). However, maltreatment was positively associated with variability within-distraction, but not the other four emotion regulation strategies, after accounting for the mean level of strategy endorsement (Table 4). That is, level of maltreatment was related to more fluctuation in using distraction from one situation to another, irrespective of whether this strategy was used more or less often across situations.

**Table 4:** The relation between childhood maltreatment and within-strategy variability (after controlling for mean strategy endorsement).

Step		Within-suppression SD			Within-distraction SD			Within-savoring SD			Within-reappraisal SD			Within-rumination SD		
		*β*	*R*^2^	∆* R*^2^	*β*	*R*^2^	∆* R*^2^	*β*	*R*^2^	∆* R*^2^	*β*	*R*^2^	∆* R*^2^	*β*	*R*^2^	∆* R*^2^
1	Intercept	0.51***	0.303	–	1.03***	0.011	–	0.65***	0.181	–	0.85***	0.043	–	0.49***	0.311	–
	Mean ER Endorsement	0.27**			0.05			0.25***			0.09*			0.26***		
2	Intercept	0.42***	0.308	0.005	0.82***	0.053	0.042*	0.82***	0.192	0.011	0.78***	0.046	0.003	0.61***	0.321	0.010
	Mean ER Endorsement	0.27*			0.04			0.24***			0.09*			0.26***		
	CTQ	0.01			0.06*			–0.01			0.01			–0.01		

SD, standard error.

#### Childhood maltreatment and emotion regulation goals

**Figure 3:**
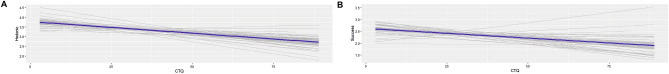
The association between childhood maltreatment and hedonic emotion regulation goals (**A**) and emotion regulation success (**B**). Abbreviation: CTQ, Childhood Trauma Questionnaire.

Childhood maltreatment was negatively associated with hedonic emotion regulation goals at the between-individual level (Table 5 and Fig. 3A). In contrast, maltreatment was positively associated with hedonic emotion regulation goals at the within-individual level. These results suggest that, when regulating emotion, people with higher levels of maltreatment are less driven by goals that focus on improving their affective state. In addition, their hedonic motivation in emotion regulation showed higher variability from one occasion to another. No significant associations were found between childhood maltreatment and instrumental goals, neither at the between-individual, nor at the within-individual level.

**Table 5:** Within-individual and between-individual relations between childhood maltreatment and emotion regulation goals.

Dimension	Parameter	Within-individual level			Between-individual level		
		Unstandardized estimate	SE	*p*	Unstandardized estimate	SE	*p*
Instrumental motives (ICC = 0.52)	Intercept	–	–	–	3.08***	0.22	0.000
	CTQ Total	0.03	0.02	0.108	− 0.11	0.09	0.268
	Residual variance	0.99***	0.00	0.000	0.98***	0.02	0.000
	*R*^2^	0.00	0.00	0.422	0.01	0.02	0.580
Hedonic motives (ICC = 0.39)	Intercept	–	–	–	3.08***	0.22	0.000
	CTQ Total	0.05**	0.09	0.007	− 0.25**	0.09	0.006
	Residual variance	0.99***	0.00	0.000	0.93	0.04	0.000
	*R*^2^	0.01	0.01	0.613	0.00	0.01	0.808

* p < .05. ** p < .01. *** p < .001.

CTQ, Childhood Trauma Questionnaire; ICC, intra-class correlation; SE, standard error.

#### Childhood maltreatment and emotion regulation success and effort

As expected, childhood maltreatment was negatively associated with emotion regulation success at both the between-individual, and the within-individual level (Table 6 and Fig. 3B). Therefore, people with higher levels of maltreatment reported less success in emotion regulation, and this pattern of reduced emotion regulation efficiency was more constant from one situation to another in them. No significant associations between maltreatment and emotion regulation effort were found, at neither level of analysis.

**Table 6:** Within-individual and between-individual relations between childhood maltreatment and emotion regulation success and difficulty.

Dimension	Parameter	Within-individual level			Between-individual level		
		Unstandardized estimate	SE	*p*	Unstandardized estimate	SE	*p*
Success (ICC = 0.29)	Intercept	–	–	–	3.82***	0.22	0.000
	CTQ Total	− 0.04*	0.02	0.038	− 0.17**	0.94	0.050
	Residual variance	0.97***	0.00	0.000	0.97***	0.03	0.000
	*R*^2^	0.01	0.00	0.299	0.03	0.03	0.365
Difficulty (ICC = 0.31)	Intercept	–	–	–	3.08***	0.22	0.000
	CTQ Total	0.01	0.02	0.311	0.05	0.09	0.626
	Residual variance	0.99***	0.00	0.000	0.99***	0.01	0.000
	*R*^2^	0.00	0.00	0.613	0.00	0.01	0.808

*p < .05. **p < .01. ***p < .001.

CTQ, Childhood Trauma Questionnaire; ICC, intra-class correlation; SE, standard error.

## Discussion

The present study offers a comprehensive description of emotion regulation in relation to history of childhood maltreatment, including differences in the use of multiple emotion regulation strategies, in emotion regulation goals, in emotion regulation success and effort. Notably, some of these differences have not been examined until now in relation to childhood maltreatment, and highlight novel aspects of emotional vulnerability in individuals with a history of maltreatment. These results also extend the limited ESM literature on childhood maltreatment, and argue for an increased focus on spontaneous emotion regulation. Some, but not all results from previous studies on habitual emotion regulation have been replicated in the present study, which is in line with the view that global and momentary measures of emotion regulation do not always overlap.

The wide range of measures allowed us to describe the relations between multiple dimensions of emotion regulation, and their association with affect. One finding was that the use of savoring was independent from or negatively correlated with the use of reappraisal, distraction, rumination and suppression. This is in line with previous studies, which have suggested that, in relation to positive emotions, savoring is used to enhance them, whereas the other four strategies are typically used to dampen them^32^. Another difference may relate to the level of engagement with emotion: savoring may involve attending and directly responding to emotion, whereas rumination, suppression, and distraction may entail avoiding emotion^49^. The present results also showed that using suppression, rumination and distraction at higher levels was associated with more negative affect, and using savoring at higher levels was associated with more positive affect (for similar results, see^49^). Hedonic and instrumental emotion regulation goals were associated with the use of similar strategies, with the exception of suppression which was related, in the present study and in previous work^43^, to instrumental goals. The pattern of self-reported emotion regulation success and effort suggested that rumination was perceived as less efficient (and indeed, that is not surprising given that, as one reviewer noted, forms of rumination such as brooding could be conceptualized as emotion regulation failures), and savoring was perceived as less effortful. Due to the relatively long intervals (i.e., up to 3 h) between assessments, we could not examine the temporal dynamics of emotion and emotion regulation. This remains a challenge for future studies, with shorter intervals between assessments, that would allow for the effect of emotion regulation at one occasion to carry over emotion at the next occasion (or vice versa).

Childhood maltreatment was associated with increased negative affect and decreased positive affect in this study. This pattern has also been reported in previous ESM studies on mood disorders and psychosis^22,50^, for instance, which underscores the potential involvement of negative and positive affect in the link between childhood maltreatment and transdiagnostic risk of psychopathology. However, not all previous studies on childhood maltreatment have replicated this pattern^28–30^. It is difficult to speculate on the possible reasons for these inconsistencies given the multiple methodological differences between studies, but it is noteworthy that the studies^25^ that found significantly increased negative affect and significantly decreased positive affect took a dimensional approach to childhood maltreatment. Dichotomous measures (e.g., checklists) and the practice of dichotomizing originally continuous maltreatment scores based on a threshold (e.g., severity) are known to reduce power and have been previously criticized^51,52^.

This study supported part of our hypotheses on emotion regulation strategies. Specifically, we expected and found that childhood maltreatment was associated with reduced use of reappraisal and savoring. Reappraisal is an emotion regulation strategy that is typically efficient in reducing negative emotions^53,54^, whereas savoring is known to efficiently increase positive emotions^55^. Therefore, lower use of these strategies in daily life may partially account for the pattern of increased negative affect and decreased positive affect that we and others have observed in association with childhood maltreatment. However, the present results failed to support the more frequent use of rumination and expressive suppression at higher levels of maltreatment. Our previous meta-analysis on habitual emotion regulation^13^ suggested that increased use of rumination and suppression are consistently associated with childhood maltreatment, and these effects are typically larger than the association with reduced reappraisal. Why, then, were we able to find only the latter effect? One explanation may be related to the characteristics of the present sample, which only included putatively healthy young adults who navigated a typical school environment throughout the sampling interval. This may have involved relatively lower levels of negative affect, which are known to promote an increased use of reappraisal^56^. Another explanation may relate to the differences between habitual and momentary measures of emotion regulation^16,17^. Future ESM studies could ideally examine the association between childhood maltreatment and use of emotion regulation strategies in representative samples of the population and in more heterogeneous contexts.

We also found that childhood maltreatment was associated with larger variability in using distraction over different contexts. A previous study^41^ found a negative association between within-strategy variability and negative affect, which suggested that using different levels of the same strategy across different occasions may be adaptive. However, this study collapsed within-strategy variability across multiple strategies, and controlled for depressive symptoms. In light of this study^41^, the present result is difficult to interpret, but it opens the way for further studies on maltreatment and emotion regulation variability and flexibility. We argue that, in light of burgeoning evidence on the association between emotion regulation flexibility and risk of psychopathology^37,39^, this topic should be increasingly investigated in research on maltreatment.

Another novel finding is that childhood maltreatment was associated, at the between-individual level, with reduced hedonic goals in emotion regulation (i.e., modulating emotion so as to feel better), and, at the within-individual level, with increased variability in hedonic emotion regulation goals. Goals are fundamental in highlighting which emotions should be modulated and in which direction (i.e., increase or decrease)^57^. For instance, when talking to a friend who has lost someone and expresses sadness, once we realize that we are also starting to experience sadness, we may choose to downregulate this emotion for hedonic reasons (e.g., feeling less bad), or upregulate it for instrumental reasons (e.g., show empathy). In the same example, if something had made us happy before we met this sad friend, we may choose to downregulate happiness or not, depending on whether we are motivated by instrumental or hedonic goals. Recent evidence suggests that emotion goals are altered in psychopathology, with depressed patients reporting that they generally want to feel less happy and more sad, and also being twice as likely to use reappraisal to increase their emotional reactions to sad pictures compared to non-depressed controls^58^. This may also be the case of individuals with a childhood maltreatment history, as suggested by the blunted motivation to regulate emotions in order to feel better. The higher variability of this motivation over time may also be related to higher positive affect variability, which has been previously reported in childhood maltreatment^30^.

The present results also show that childhood maltreatment is associated with reduced perceived emotion regulation success in daily life. This in in line with previous evidence from studies using dispositional (habitual) measures, which have indicated a consistent association between childhood maltreatment and increased self-reported emotion regulation difficulties (as well as decreased emotion regulation abilities)^13^. Emotion regulation effort was not related to childhood maltreatment in this study. A previous study^6^ suggested that individuals with a history of maltreatment may expend increased effort when using reappraisal, but this was based on neural activity rather than subjective reports and it involved a laboratory task in which participants were instructed to use reappraisal (i.e., reappraisal ability) rather than spontaneously using reappraisal, as in this study.

The main limitation of this study is related to the focus on a student sample, which puts into question the generalizability of these results. The reason for choosing this sample was to maximize compliance with the intensive 10-day ESM design. Another reason was that this sample was followed up on the occasion of an exam, with the aim of investigating the relations between spontaneous emotion regulation in daily life and during acute stress (these data will be reported in another manuscript). Generalizability is also limited by the asymmetric sex distribution, with most of the present sample being women. Another limit, in our opinion, is related to not having collected data about the contexts. Previous studies^59,60^ have assessed daily events and found greater emotional reactivity in childhood maltreatment, with both higher negative affect and positive affect following negative and positive events, respectively. Our focus (and indeed, one of the sources of novelty in this study) was on emotion regulation, and we chose to include more items on the characteristics of this domain. ESM questionnaires need to be kept as short as possible in order to limit the interruption of current activities^24^. It is noteworthy that, for theoretical reasons discussed elsewhere^13,61^, we chose to focus on cumulative childhood maltreatment, rather than examine the putatively specific effects of different forms of maltreatment. Briefly, in light of evidence of significant co-occurrence of multiple forms of maltreatment, disentangling their effects seems empirically unrealistic.

In conclusion, these results indicate that childhood maltreatment is associated with emotion regulation differences in daily life, at multiple levels including strategy selection, goals, and overall efficiency. They support the view that emotion regulation is one of the important domains that could shed light on the mechanisms between childhood maltreatment and risk of psychopathology. We argue that focusing on process measures of emotion regulation is crucial for identifying targets for the next generation of mental health interventions in childhood maltreatment.

## Methods

### Participants

The present sample included N = 118 participants (82.76% women; age M = 19.65, range 18–33 years). They were all students enrolled in the same course. We did not run an a priori sample size estimation. However, it is noteworthy that there is little consensus on the methods of estimating sample size a priori in multilevel models^62–64^, and current recommendations rely on common practice in previous studies. For instance, Gabriel and colleagues^62^ reviewed sample size in over 100 ESM studies published in top-tier journals. They found that the mean level 2 (between-individual) sample size was 83, and the mean level 1 (within-individual) sample size was 835, and recommended that studies should aim for at least these sample sizes. The present sample is comparable to those in other ESM studies, and exceed the suggested sample size necessary for Level 1 and Level 2 inferences^62^. Before the beginning of the study, participants were informed about the procedure and signed an informed consent. All measures were collected, stored and analyzed anonymously, in line with the Declaration of Helsinki recommendations. The study was approved by the Ethical Committee of Babeș-Bolyai University, as part of the co-first author (M.I.B.) Ph.D. thesis, under the supervision of the senior author (A.C.M.). Course credit was provided for participation in this study.

### Measures

#### Childhood maltreatment

Childhood maltreatment was assessed using the short form of the Childhood Trauma Questionnaire (CTQ)^65,66^. This scale includes 25 items (rated on 5-point Likert scale ranging from 1 = never true to 5 = very often true) assessing multiple forms of abuse and neglect while the respondent was growing up: (1) emotional abuse (i.e., “verbal assaults on a child’s sense of worth or well-being or any humiliating or demeaning behavior directed toward a child by an adult or older person”); (2) sexual abuse (i.e., “sexual contact or conduct between a child younger than 18 years of age and an adult or older person”); (3) physical abuse (i.e., “bodily assaults on a child by an adult or older person that posed a risk of or resulted in injury”); (4) emotional neglect (i.e., “the failure of caretakers to meet children’s basic emotional and psychological needs, including love, belonging, nurturance, and support”); and (5) physical neglect (i.e., “the failure of caretakers to provide for a child’s basic physical needs, including food, shelter, clothing, safety, and health care”)^65,^ ^p. 175^. A total childhood maltreatment score was calculated by summing the five subscale scores, showing good reliability in the present sample (Cronbach’s alpha = 0.89).

#### Experience sampling questionnaire

The ESM questionnaire was adapted from established emotion (e.g., Positive and Negative Affect Schedule^67^) and emotion regulation (e.g., Emotion Regulation Questionnaire^68^; Response Style Questionnaire^69^) scales, and included items related to: (1) positive (i.e., happiness/joy, surprise, pride) and negative (i.e., anger, sadness, fear/anxiety, shame, guilt, boredom, disgust) affect (*how did you feel since the last beep?*), rated on 5-point Likert scale (ranging from 0 = not at all, to 5 = very much); (2) emotion regulation strategies (*what did you do to try to change your emotions?*), including: (*i*) distraction (*I tried to redirect my attention to something else*)*,* (*ii*) savoring (*I tried to savor my emotions as much as possible*), (*iii*) cognitive reappraisal (*I tried to see the situation in a different way*), (*iv*) rumination/reflection (*I tried, but I couldn’t stop thinking about what happened*), and (*v*) expressive suppression (*I tried not to show my emotions to others*), all rated on a 5-point Likert scale (ranging from 0 = not at all, to 5 = very much); (3) emotion regulation goals (*why did you try to change your emotions?*): hedonic (1 item: *to feel better*) and instrumental (3 items: *to maintain appearances*; *to make others feel better*; *to focus on what I needed to do*), all rated on a 5-point Likert scale (ranging from 0 = total disagreement, to 5 = total agreement); (4) emotion regulation success (*how well did you succeed to change your emotions?*), rated on a 5-point Likert scale from 0 = not at all, to 5 = very well); and (5) emotion regulation effort (*how hard was it to change your emotions?*), rated on a 5-point Likert scale from 0 = not at all, to 5 = very much). In the case of items with multiple answers (i.e., affect, emotion regulation strategies, emotion regulation goals), all answers were rated.

### Procedure

The study took place during the course of the semester, in November 2018, at Babeș-Bolyai University. The Personal Analytic Companion (PACO) mobile app was used to schedule and administer questionnaires. Before the study, participants attended a training session in which questionnaire items (e.g., construct definitions, response options) and the procedure were carefully explained. Once the study started, the app prompted participants 3 times per day, for 10 consecutive days, including workdays and weekends. Notifications were sent at random times between 10 AM and 8 PM, with a minimum of 60 min between notifications. At each sampling moment, participants were directed to a short questionnaire assessing multiple characteristics of emotions (i.e., emotional experience, the use of emotion regulation strategies, emotion regulation goals, overall emotion regulation efficiency and effort) that occurred in the time interval since the last notification. After each notification, participants had 20 min to respond, otherwise the response was registered as missing data.

### Statistical analysis

In order to describe childhood maltreatment, we categorized CTQ scores based on the severity cutoffs reported in the scale manual^66^. Notably, the low-to-moderate cutoff has the highest sensitivity, while keeping the specificity level to an acceptable level (> 80%). This cutoff allows one to identify participants with any level of maltreatment, which is in line with the goals of the present study. However, continuous CTQ scores were used in the main analysis.

To exploit the hierarchical structure of the data (three daily measurement occasions nested into persons), we employed a multilevel modeling technique. This analysis is effective in disentangling between- and within-individual effects, and in handling missing data^70^. Grand-mean centering was employed. Following the approach outlined by Kleiman^71^, first, we first ran unconditional models (“intercept-only” models) in order to ensure that there is sufficient between-participant supporting the deployment of multi-level modeling as our main data analysis approach. The corresponding ICCs derived via the unconditional models are reported in Tables 2–3 and 5–6. Next, we ran a two-level random intercept model, where childhood maltreatment predicted the various outcomes pertaining to affect or to emotion regulation: positive affect, negative affect, emotion regulation strategies, emotion regulation goals, and emotion regulation success and effort. An additional outcome was emotion regulation variability, as reflected by two indices calculated based on the standard deviations of the emotion regulation strategy items: the between-strategy variability (i.e., variation in the level of using multiple strategies at each measurement occasion) and the within-strategy variability (i.e., variation in the level of using the same strategy over different contexts and time). All the formulas are presented in Blanke et al.^41^, and have been used without modification. The only exception is that instead of calculating a global within-strategy variability across all strategies, we calculated this index for each separate strategy. As recommended^41^, we controlled for the mean endorsement level of the corresponding strategy in all analyses focused on within-strategy variability. All the paths between the independent (childhood maltreatment) and the dependent variables (positive/negative affect, emotion regulation strategies, emotion regulation variability, emotion regulation goals, and emotion regulation success and effort) were estimated at both levels of analysis (within- and between-individual). We report standardized parameter estimates in order to indicate how much of the actual level of change in the outcome (e.g., emotion regulation) variables corresponds to the change in childhood trauma (measured in standard deviations). All the estimators were derived by employing maximum likelihood estimator. Analyses were conducted using Mplus version 7^72^.

## Supplementary Information

Supplementary Information.

## Data Availability

The data analyzed in current study are not publicly available due the fact that they are part of a larger dataset that is subject to other ongoing analyses (with a different focus), but are available from the corresponding authors on reasonable request.
