# Supplementary material for: Childhood maltreatment and emotion regulation in everyday life: an experience sampling study
Source: Sci Rep. 2023 May 3;13:7214. doi: 10.1038/s41598-023-34302-9 (PMC10156801; doi:10.1038/s41598-023-34302-9)
Supplement: Supplementary file 1 — Supplementary Information. [file 41598_2023_34302_MOESM1_ESM.docx]

**Supplementary Tables**

**Supplementary Table 1**

Within-individual and between-individual relations among emotion regulation strategies

| Between-individual  Within-  individual | Suppression | Distraction | Savoring | Reappraisal | Rumination |
| --- | --- | --- | --- | --- | --- |
| Suppression | - | 0.65^***^ | 0.05 | 0.46^***^ | 0.56^***^ |
| Distraction | 0.39^***^ | - | -0.04 | 0.69^***^ | 0.54^***^ |
| Savoring | -0.19^***^ | -0.28^***^ | - | 0.12 | 0.04 |
| Reappraisal | 0.25^***^ | 0.42^***^ | -0.16^***^ | - | 0.62^***^ |
| Rumination | 0.26^***^ | 0.26^***^ | -0.16^***^ | 0.26^***^ | - |

*Note:* **** p < .001*.

**Supplementary Table 2**

Within-individual and between-individual relations between emotion regulation strategies and affect

|  | | Within-individual level | | | Between-individual level | | |
| --- | --- | --- | --- | --- | --- | --- | --- |
| Outcome | Parameter | Unstandardized estimate | SE | *p* | Unstandardized estimate | SE | *p* |
| Negative Affect | Intercept | - | - | - | -2.15 | 0.19 | 0.000 |
|  | Suppression | 0.11 | 0.02 | 0.000 | 0.24 | 0.11 | 0.033 |
|  | Distraction | 0.03 | 0.02 | 0.028 | 0.32 | 0.16 | 0.045 |
|  | Savoring | -0.24 | 0.02 | 0.000 | -0.06 | 0.08 | 0.446 |
|  | Reappraisal | 0.05 | 0.02 | 0.000 | -0.11 | 0.15 | 0.445 |
|  | Rumination | 0.38 | 0.02 | 0.000 | 0.29 | 0.10 | 0.006 |
|  | Residual variance | 0.68 | 0.02 | 0.000 | 0.56 | 0.07 | 0.000 |
|  | *R^2^* | 0.32 | 0.01 | 0.000 | 0.44 | 0.07 | 0.000 |
| Positive Affect | Intercept | - | - | - | -3.29 | 0.33 | 0.000 |
|  | Suppression | 0.03 | 0.02 | 0.029 | 0.06 | 0.10 | 0.524 |
|  | Distraction | -0.04 | 0.02 | 0.012 | -0.24 | 0.14 | 0.099 |
|  | Savoring | 0.59 | 0.01 | 0.000 | 0.70 | 0.05 | 0.000 |
|  | Reappraisal | -0.00 | 0.02 | 0.713 | 0.28 | 0.13 | 0.039 |
|  | Rumination | -0.07 | 0.02 | 0.000 | 0.05 | 0.09 | 0.551 |
|  | Residual variance | 0.62 | 0.01 | 0.000 | 0.39 | 0.33 | 0.000 |
|  | *R^2^* | 0.38 | 0.01 | 0.000 | 0.60 | 0.07 | 0.000 |

Abbreviations: SE, standard error.

**Supplementary Table 3**

Within-individual and between-individual relations between emotion regulation strategies and emotion regulation goals

|  | | Within-individual level | | | Between-individual level | | |
| --- | --- | --- | --- | --- | --- | --- | --- |
| Outcome | Parameter | Unstandardized estimate | SE | *p* | Unstandardized estimate | SE | *p* |
| Hedonic goals | Intercept | - | - | - | -2.81 | 0.38 | 0.000 |
|  | Suppression | 0.01 | 0.02 | 0.545 | -0.06 | 0.11 | 0.594 |
|  | Distraction | 0.20 | 0.02 | 0.000 | 0.16 | 0.16 | 0.311 |
|  | Savoring | 0.06 | 0.01 | 0.000 | 0.14 | 0.08 | 0.089 |
|  | Reappraisal | 0.24 | 0.02 | 0.000 | 0.48 | 0.14 | 0.001 |
|  | Rumination | 0.09 | 0.02 | 0.000 | 0.09 | 0.10 | 0.379 |
|  | Residual variance | 0.83 | 0.01 | 0.000 | 0.54 | 0.07 | 0.000 |
|  | *R^2^* | 0.17 | 0.01 | 0.000 | 0.46 | 0.07 | 0.000 |
| Instrumental goals | Intercept | - | - | - | -3.81 | 0.29 | 0.000 |
|  | Suppression | 0.30 | 0.02 | 0.000 | 0.51 | 0.11 | 0.033 |
|  | Distraction | 0.19 | 0.02 | 0.000 | 0.16 | 0.16 | 0.045 |
|  | Savoring | 0.02 | 0.02 | 0.089 | 0.13 | 0.08 | 0.446 |
|  | Reappraisal | 0.18 | 0.02 | 0.000 | 0.21 | 0.15 | 0.445 |
|  | Rumination | 0.05 | 0.02 | 0.002 | 0.16 | 0.10 | 0.006 |
|  | Residual variance | 0.72 | 0.01 | 0.000 | 0.19 | 0.04 | 0.000 |
|  | *R^2^* | 0.28 | 0.01 | 0.000 | 0.81 | 0.03 | 0.000 |

**Supplementary Table 4**

Within-individual and between-individual relations between emotion regulation strategies and emotion regulation success and effort

|  | | Within-individual level | | | Between-individual level | | |
| --- | --- | --- | --- | --- | --- | --- | --- |
| Outcome | Parameter | Unstandardized estimate | SE | *p* | Unstandardized estimate | SE | *p* |
| Success | Intercept | - | - | - | -3.07 | 0.38 | 0.000 |
|  | Suppression | 0.07 | 0.02 | 0.000 | 0.03 | 0.12 | 0.033 |
|  | Distraction | 0.22 | 0.02 | 0.000 | 0.38 | 0.16 | 0.045 |
|  | Savoring | 0.15 | 0.02 | 0.000 | 0.25 | 0.08 | 0.446 |
|  | Reappraisal | 0.21 | 0.02 | 0.000 | 0.34 | 0.15 | 0.445 |
|  | Rumination | -0.11 | 0.02 | 0.000 | -0.20 | 0.11 | 0.006 |
|  | Residual variance | 0.85 | 0.01 | 0.000 | 0.19 | 0.04 | 0.000 |
|  | *R^2^* | 0.14 | 0.01 | 0.000 | 0.44 | 0.07 | 0.000 |
| Effort | Intercept | - | - | - | -2.12 | 0.39 | 0.000 |
|  | Suppression | 0.10 | 0.02 | 0.000 | -0.02 | 0.10 | 0.811 |
|  | Distraction | 0.14 | 0.02 | 0.000 | 0.46 | 0.15 | 0.002 |
|  | Savoring | -0.19 | 0.02 | 0.000 | -0.10 | 0.07 | 0.166 |
|  | Reappraisal | 0.08 | 0.02 | 0.000 | -0.22 | 0.14 | 0.119 |
|  | Rumination | 0.27 | 0.02 | 0.000 | 0.57 | 0.09 | 0.000 |
|  | Residual variance | 0.72 | 0.01 | 0.000 | 0.44 | 0.07 | 0.000 |
|  | *R^2^* | 0.28 | 0.01 | 0.000 | 0.55 | 0.07 | 0.000 |
